# ERG-Associated lncRNA (ERGAL) Promotes the Stability and Integrity of Vascular Endothelial Barrier During Dengue Viral Infection via Interaction With miR-183-5p

**DOI:** 10.3389/fcimb.2020.00477

**Published:** 2020-09-08

**Authors:** Baojia Zheng, Hui Wang, Guohui Cui, Qianfang Guo, Lulu Si, Huijun Yan, Danyun Fang, Lifang Jiang, Zhenyou Jiang, Junmei Zhou

**Affiliations:** ^1^Department of Microbiology and Immunology, School of Basic Medical Sciences, Jinan University, Guangzhou, China; ^2^Key Laboratory of Tropical Disease Control, Ministry of Education, Sun Yat-sen University, Guangzhou, China; ^3^Department of Medical Microbiology, Zhongshan Medical College, Sun Yat-sen University, Guangzhou, China

**Keywords:** lncRNA, ERGAL, dengue virus, severe dengue infection, permeability, miRNA-microRNA

## Abstract

Dengue virus (DENV) continues to be a major public health problem. DENV infection will cause mild dengue and severe dengue. Severe dengue is clinically manifested as serious complications, including dengue hemorrhagic fever and/or dengue shock syndrome (DHF/DSS), which is mainly characterized by vascular leakage. Currently, the pathogenesis of severe dengue is not elucidated thoroughly, and there are no known therapeutic targets for controlling the disease effectively. This study aimed to further reveal the potential molecular mechanism of severe dengue. In this study, the long non-coding RNA, ERG-associated lncRNA (lncRNA-ERGAL), was activated and significantly up-regulated in DENV-infected vascular endothelial cells. After knockdown of lncRNA-ERGAL, the expression of ERG, VE-cadherin, and claudin-5 was repressed; besides, cell apoptosis was enhanced, and cytoskeletal remodeling was disordered, leading to instability and increased permeability of vascular endothelial barrier during DENV infection. Fluorescence *in situ* hybridization (FISH) assay showed lncRNA-ERGAL to be mainly expressed in the cytoplasm. Moreover, the expression of miR-183-5p was found to increase during DENV infection and revealed to regulate ERG, junction-associated proteins, and the cytoskeletal structure after overexpression and knockdown. Then, ERGAL was confirmed to interact with miR-183-5p by luciferase reporter assay. Collectively, ERGAL acted as a miRNA sponge that can promote stability and integrity of vascular endothelial barrier during DENV infection via binding to miR-183-5p, thus revealing the potential molecular mechanism of severe dengue and providing a foundation for a promising clinical target in the future.

## Introduction

Dengue virus (DENV) is a single positive-strand RNA virus belonging to *Flaviviridae* family, and is spread by *Aedes* mosquitoes. DENV has four virus serotypes (DENV-1–4), all circulating worldwide, including in Asia, Africa, and the Americas. A total of 390 million cases of dengue infection are estimated to occur per year, of which 96 million clearly show severe clinical or subclinical manifestations, ranging from mild fever to potentially fatal syndromes such as dengue hemorrhagic fever and dengue shock syndrome (DHF/DSS) (Guzman et al., 2010; Bhatt et al., 2013), which is the most severe complication of DENV infection. Severe dengue, referred to as the occurrence of DHF/DSS, whose main pathological feature and clinical manifestation is the increased vascular permeability, results in the paracellular leakage of plasma fluids and protein and exhibits clinical symptoms like bleeding or diffuse intravascular coagulation and subsequently death. It is estimated that the mortality rate can be as high as ~50% in infants or secondary heterotypic infections (Martina et al., 2009). However, the pathogenesis of severe dengue has not been fully elucidated to date.

Vascular endothelial cells form a vascular barrier to prevent blood cells from extravasation and maintain a dynamically stable tissue environment. However, the paracellular barrier is the preferred route, when cells and solutes migrate or leak from the blood vessels due to pathophysiological changes (Vestweber et al., 2009). The paracellular barrier, which is also defined as the connection between endothelial cells, is mainly formed by adherent junctions and tight junctions that dynamically regulate permeability of the endothelium (Aghajanian et al., 2008). Adherent junctions, composed of transmembrane vascular endothelial (VE)-cadhesions, can interact with VE-cadherin expressed in adjacent cells via a homotropic mechanism to regulate paracellular permeability (Vestweber, 2008). Claudin-5, a member of the claudin family constituting the tight junctions, is enriched specifically in endothelial cells, where its induced expression alone is sufficient to reconstitute the paracellular barrier and block large molecules from entering the leaky endothelial cells (Morita et al., 1999; Soma et al., 2004; Aghajanian et al., 2008; Vandenbroucke et al., 2008). Besides, expression and clustering of VE-cadherin at junctions are required to up-regulate the transcription of claudin-5 (Taddei et al., 2008), all mentioned above revealing that both VE-cadherin and claudin-5 are key junctional proteins in cell junctions, playing crucial roles in regulating vascular permeability.

Vascular development and angiogenesis require the ETS transcription factor family (Randi et al., 2009). ETS-related gene (ERG) was found to express in the endothelium and regulates vascular homeostasis, angiogenesis, and stability, as well as monolayer integrity and cell growth (Birdsey et al., 2012, 2015). Moreover, it is also involved in modulating permeability and cell survival by regulating the transcription of VE-cadherin or claudin-5 (Birdsey et al., 2008; Yuan et al., 2012). In other words, cytoskeleton and associated proteins can also function as effective regulatory elements of the endothelial barrier (Wang et al., 2010), such as F-actin, which is also known to be particularly important in maintaining integrity and regulating epithelial junction remodeling, affecting the permeability of tissue barriers (Wang et al., 2015).

Only 2% of human genes can encode proteins, while the rest consist of transcriptional regulatory elements and non-coding RNAs. Of them, long non-coding RNAs (lncRNAs) are a kind of non-coding RNAs with at least 200 nucleotides, accounting for ~80% of all non-coding RNAs (Collins, 2004; Costa, 2005; The FANTOM Consortium, 2005). LncRNAs reportedly play important roles in regulating chromatin remodeling, controlling gene transcription, participating in mRNA transcription, regulating protein function, and participating in intercellular signaling (Mercer et al., 2009). The viruses, like human immunodeficiency viruses, hepatitis B virus, influenza virus, and Epstein–Barr virus, could induce dysregulated expression of lncRNAs, thereby altering the normal function of the host cell. Such differentially expressed lncRNAs are shown to regulate cell proliferation and differentiation as well as to be involved in innate immunity and signal transduction for regulating viral infection (Iwakiri and Takada, 2010; Peng et al., 2010; Lau et al., 2014; Saayman et al., 2014). What is more, lncRNA is reported to localize to the nucleus and/or cytoplasm, and their subcellular localization pattern is considered to indicate their biological basis and potential molecular roles (Derrien et al., 2012; Fort et al., 2014). A new regulatory mechanism has been suggested, involving cytoplasmic lncRNAs that act as natural microRNA sponges to interfere with miRNA pathways, thus reducing the binding of endogenous miRNAs to target genes for fulfilling their functions at post-transcriptional levels (Quinn and Chang, 2015).

In our previous study, monolayers of primary endothelial cells had been used as a model for analyzing the function of the endothelial barrier during DENV infection. We had reported that DENV infection could induce a mass of differentially expressed lncRNAs in vascular endothelial cells; furthermore, the corresponding target genes predicted were mainly involved in important biological processes closely related to severe dengue, hence indicating that differentially expressed lncRNAs were potentially related to the regulation of pathology of severe dengue (Zheng et al., 2019). Of the differentially expressed lncRNAs, the lncRNA with non-code transcript ID: NONHSAT190967 and non-code gene ID: NONHSAG082924 (Supplementary Table 1) was found to be increased during viral infection and was located downstream of the transcription factor ERG (GenBank:NM_182918) in cis-regulation. Since *ERG* is associated with vascular permeability, we regarded this as the putative lncRNA that might be associated with vascular permeability. Measurements with respect to lncRNA and ERG were conducted, and a strong correlation was identified; therefore, we proposed its name as “*ERG*-associated lncRNA (ERGAL),” considering its special location and strong correlation. Functional experiments were conducted thereafter, and lncRNA-ERGAL was found to be involved in the regulation of endothelial barrier permeability during DENV infection by binding to miR-183-5p.

The present study highlights an important role of lncRNA-ERGAL in promoting integrity and stability of endothelial barrier during DENV infection and in the significant interaction between lncRNA-ERGAL and miR-183-5p as a regulator, which might be considered a promising target for treating severe dengue in future.

## Materials and Methods

### Cell Culture and Viral Infection

*Aedes albopictus* mosquito (C6/36) cells were preserved in our laboratory and were cultured in minimum essential medium (MEM) (Thermo Scientific, CA, USA) with 8% fetal calf serum (Gibco, Carlsbad, CA, USA) and 0.1% penicillin–streptomycin solution (Gibco) at 28°C. Human umbilical vascular endothelial cells (HUVECs) were obtained from ScienCell Research Laboratories (Carlsbad, CA, USA) and cultured using the ECM Bullet Kit (ScienCell Research Laboratories) in an incubator (Thermo Scientific, USA) at 37°C and 5% CO_2_.

DENV-I strain GD/FS/01/2014 was propagated in C6/36 cells, and virus titers were measured using the TCID_50_ assay. HUVECs were cultured in 6-, 24-, and 96-well plates for infection with or without transfection for 24 h. The infection procedure included addition of DENV at the desired multiplicity of infection (MOI) followed by incubation for 2 h at 37°C. The following assays were conducted at 24 h post-infection. Cells were also incubated with the culture supernatant as mock-infected control.

### Quantitative Reverse Transcription PCR (qRT-PCR)

After the intervention, total RNA was extracted using Trizol reagent (Invitrogen, Carlsbad, CA, USA) according to the manufacturer's protocol, followed by quantitative and qualitative RNA analyses using the NanoDrop 2000 (Thermo Scientific, USA). RNA was reverse transcribed to cDNA using the PrimeScript RT Master Mix kit (Takara, Japan) according to the manufacturer's instructions for detecting the expression of ERGAL, VE-cadherin, and claudin-5, while the Evo M-MLV RT Kit (Accurate Biotechnology, China) was used for analyzing microRNA. qPCR was performed using the TB Green Premix Ex TaqII kit (Takara, Japan) on a CFX96 Real-Time PCR system (BIO-RAD, USA). The relative expression of lncRNA, mRNA, or miRNA was calculated using the comparative ΔΔCt method. Primer sequences used for the qRT-PCR assay are provided in Supplementary Table 2.

### Western Blotting

Cultured cells (5 × 10^6^) were lysed on ice using PMSF lysate buffer. Samples containing 40–60 μg of protein were subjected to thermal denaturation and SDS-PAGE and were then transferred to PVDF membranes. After the membrane was sealed with a blocking solution (TBST containing 5% skim milk) for 60 min, it was incubated with the primary antibody: anti-ERG (Abcam, England, Ca#133264), anti-VE-cadherin (Abcam, Ca# ab33168), and anti-claudin-5 (Abcam, Ca# ab15106) overnight and then with the HRP-labeled secondary antibody (Proteintech, USA, Ca# SA00001-1) at 37°C or 1 h. Finally, the BeyoECLPlus chemiluminescence reagent (Beyotime, China, P0018) was used to visualize the protein bands.

### Immunofluorescence Microscopy

Huvecs were washed once using phosphate-buffered saline (PBS; pH 7.4), fixed with 4% paraformaldehyde for 20 min, and blocked with PBS containing 10% goat serum, 0.3 M glycine, 1% BSA, and 0.1% Tween for 1 h at room temperature. Cells were incubated with the following primary antibodies: anti-VE-cadherin antibody (Abcam, England, Ca# ab33168) at a 1/500 dilution and anti-claudin-5 antibody (Abcam, Ca# ab15106) at a 1/200 dilution at 4°C overnight. Cells were then washed with PBS-Tween-20 (PBST) three times and incubated with fluorophore-conjugated secondary antibodies at a 1/100 dilution for 1 h at room temperature. DAPI was used to stain the cell nuclei for 5 min. Samples were imaged on an Axio Imager Z1 (ZEISS, Germany).

### Cytoskeletal Staining

Phalloidin-fluorescein isothiocyanate (Sigma-Aldrich, Ca# P5282) was used to label actin filaments. Cells were washed with PBS and fixed with 3.7% formaldehyde solution in PBS for 5 min and again washed extensively in PBS. Cells were then permeabilized with 0.1% Triton X-100 in PBS and stained with a 50 mg/ml fluorescent phalloidin conjugate solution in PBS (containing 1% DMSO from the original stock solution) for 40 min at room temperature. The samples were washed several times with PBS to remove unbound phalloidin conjugate. Samples were imaged on an Axio Imager Z1 (ZEISS, Germany).

### Transfection

Transfections were performed using Lipofectamine RNAiMAX (Invitrogen Corporation, Carlsbad, CA, USA) according to the manufacturer's instructions. Short interfering (siRNA) targeting lncRNA-ERGAL and miR-183-5p inhibitors were transfected into HUVECs at a final concentration of 100 μM, whereas themiR-183-5p mimic was introduced at a final concentration of 50 μM. si-lncRNA, si-NC, miR-183-5p mimic, miR-183-5p inhibitor, and miR-NC were all designed and synthesized by RiboBio (RiboBio Co., Guangzhou, China) (Supplementary Table 3). Cells were then infected with DENV at 24 h after transfection.

### Apoptosis Assay

Apoptosis was detected by flow cytometry using the Annexin V-FITC/PI cell apoptosis detection kit (Transgen biotech, Guangzhou, China). After stimulating the cells, they were washed twice in PBS before being resuspended in 100 μl of binding buffer and 5 μl of Annexin V-FITC; then, 5 μl of PI was added in dark conditions to stain the cell for 15 min according to the manufacturer's instructions.

### Transwell Assay

An FITC-dextran/transwell assay was used to assess monolayer cell permeability, as described previously (Sedgwick et al., 2002; Irwin et al., 2005). HUVECs were seeded in the upper chamber. After transfection and viral stimulation, the culture medium of the upper chamber was switched to a non-serum culture medium with 5 μg/ml FITC-dextran (Chondrex, Washington, USA, Ca #4009). A culture medium containing 5% FBS was added to the lower chamber and incubated for 1 h at 37°C. The fluorescence energy value (with an excitation wavelength of 494 nm and an emission wavelength of 520 nm) was detected by SpectraMaxM5 (Molecular Devices, USA). FITC-dextran fluorescence energy values from the upper and lower compartments were obtained; further, the permeability coefficient of dextran (Pd) in HUVECs was calculated according to the following formula: Pd (cm/s) = ([*A*]/*t*) × (*I*/*A*) × (*V*/[*L*]), where [*A*] is the FITC-dextran fluorescence energy value of the lower chamber, *t* (s) is the time interval, *A* (cm^2^) is the transwell surface area, *V* (m^3^) is the volume of the solution in the lower chamber, and [*L*] is the fluorescence energy value of FITC-dextran in the upper chamber. The ratio of Pd corresponds to either the si-lncRNA group or si-NC group divided by the NC group.

### Fluorescence *in situ* Hybridization (FISH)

The subcellular localization of lncRNA in DENV-infected HUVECs was identified by a FISH assay. The lncRNA-ERGAL probe for the assay was designed, and the protocol was conducted using the *in situ* Hybridization kit (Exon Biotechnology, Guangzhou, China) according to the manufacturer's instructions. Images were acquired by laser scanning confocal microscopy LSM710 (ZEISS, Germany).

### Double Luciferase Reporter

The mutated lncRNA-ERGAL sequence comprising seven mutated nucleotides within the miR-183-5p binding sites was inserted into a vector. Each construct was co-transfected with the indicated miRNAs (RiboBio Co., Guangzhou, China) into 293T cells using Lipofectamine 3000 (Invitrogen, Carlsbad, CA, USA) for 48 h. Luciferase assays were performed using the Dual-Luciferase Reporter Assay System (Promega, WI, USA) according to the manufacturer's instructions. Luminescence signals were quantified using the BioTek Synergy HTX multi-mode reader, and luciferase activity was presented as the relative hRluc/hluc ratio.

### Statistical Analysis

All data analyses were carried out using SPSS20.0 statistical software. The data were presented as the means ± standard deviation (SD) of at least three independent experiments. Comparison between the two groups was made using the Student's *t*-test. Differences were considered statistically significant when *P* < 0.05.

## Results

### Expression of lncRNA-ERGAL Is Induced by DENV in a Time-Dependent Manner

**Figure 1:**
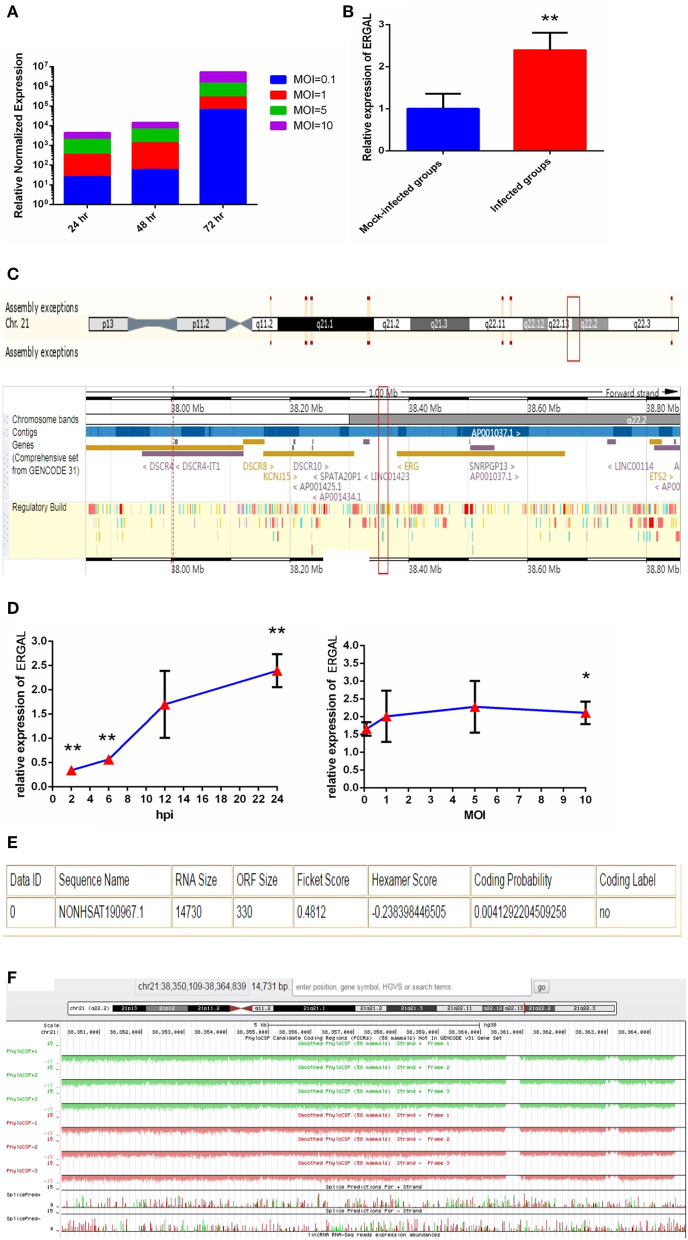
The expression of ERGAL was up-regulated after DENV infection. **(A)** The viral load in HUVECs showed to be in dose-dependent and time-dependent manner. The expression of E gene of dengue virus in HUVECs was detected by RT-qPCR at different infectious MOIs (a MOI from 0.1 to 10) and time intervals (24 hpi, 48 hpi, and 72 hpi) (*n* = 3) compared with those mock-infected groups. **(B)** The expression of ERGAL was increased after DENV infection. The expression levels of ERGAL were detected by RT-qPCR in HUVECs infected with DENV(MOI = 10, 24 hpi). Values were means ± SD (*n* = 6). **P < 0.01 vs. mock-infected control group. **(C)** ERGAL genome was located on chromosome 21(start site from 38350109 to end site 38364839) predicted at Ensembl website (http://asia.ensembl.org/index.html). **(D)** The expression pattern of ERGAL was in a time-dependent manner but virus dose-independent. The expression levels of ERGAL were assessed in HUVECs by RT-qPCR at different infectious time intervals (2hpi, 6 hpi, 12 hpi, and 24 hpi with MOI = 10) or different infectious MOIs (MOI = 0.1, 1, 5, and 10 with 24 hpi). Values were means ± SD (*n* = 4). *P < 0.05, **P < 0.01 vs. uninfected control groups. **(E,F)** ERGAL had no protein-coding potential. The bioinformatics prediction of coding protein function of lncRNA-ERGAL was analyzed with Coding-Potential Assessment Tool (http://lilab.research.bcm.edu/cpat/index.php) and PhyloCSF output value assessment at UCSC Genome Browser Gateway (http://genome-asia.ucsc.edu/cgi-bin/hgGateway). The experiments were performed independently at least three times with similar results.

Since the titer peak of viremia in the early stages of infection is closely correlated with the severity of DENV infection in humans (Vaughn et al., 2000), we performed a qRT-PCR assay to measure the viral load in HUVECs subjected to different virus doses and infectious periods to optimize the infectious dose and duration of infection. We found the viral load in HUVECs to increase notably from 24 to 72 h post-infection, and it tended to increase with MOI values ranging from 0.1 to 10 (Figure 1A). An MOI value of 10 could acquire the peak viral load in HUVECs. Owing to the rapid growth of cells and dynamic viral load, we identified infected HUVECs with an MOI value of 10 at 24 h post-infection as a model for conducting high-throughput sequence analyses regarding severe dengue.

Sequence analysis revealed that DENV infection could induce a mass of differentially expressed lncRNAs, of which lncRNA-ERGAL was specifically activated and overexpressed post-infection compared to that in the mock-infected control groups. qRT-PCR was used to verify the expression of ERGAL. As shown in Figure 1B, expression of ERGAL was up-regulated after DENV infection. Bioinformatics analysis showed ERGAL to be located on chromosome 21, from site 38,350,109 to 38,364,839, within 20-kb downstream of the transcription factor *ERG* (Figure 1C). ERGAL was found to be induced specifically by DENV, suggesting the possible association of ERGAL with the pathology of virus infection. The expression pattern of ERGAL was explored further and found to be increased significantly between 2 and 24 h post-infection; however, it was unrelated to the viral dose administered (Figure 1D), indicating that the up-regulation of ERGAL occurred in a time-dependent manner (from 2 to 24 h post-infection) rather than dose-dependent. Analysis using the Coding-Potential Assessment Tool (Wang et al., 2013) and PhyloCSF (Lin et al., 2011) indicated this lncRNA to have no protein-coding potential (Figures 1E,F).

### LncRNA-ERGAL Was Correlated With the Expression of ERG

**Figure 2:**
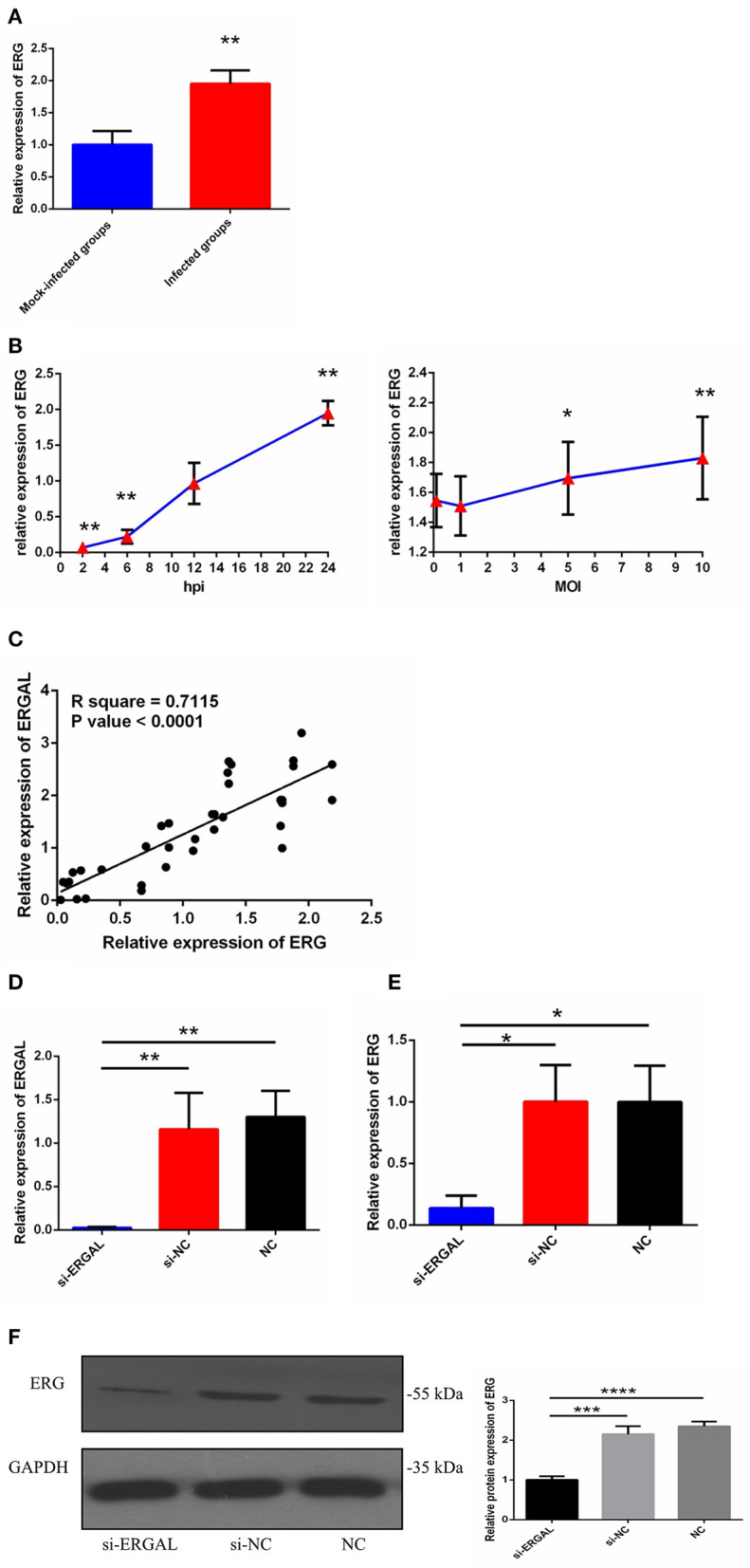
LncRNA-ERGAL was correlated with ERG expression. **(A)** The expression of ERG was up-regulated after DENV infection. The expression levels of ERG were detected in HUVECs by RT-qPCR (MOI = 10, 24 hpi). Values were means ± SD (*n* = 6). **P < 0.01 vs. mock-infected groups. **(B)** The expression pattern of ERG was in a time-dependent manner but virus dose-independent. The expression levels of ERG were assessed in HUVECs by RT-qPCR at different infectious time intervals (2 hpi, 6 hpi, 12 hpi, and 24 hpi with MOI = 10) or at different infectious MOIs (MOI = 0.1, 1, 5, and 10 with 24 hpi). Values were means ± SD (*n* = 4). *P < 0.05, **P < 0.01 vs. uninfected group. **(C)** ERGAL was strongly correlated with ERG expression. The relationship between ERGAL and ERG was assessed by Pearson correlation analysis. **(D)** The expression of ER GAL was remarkably inhibited after DENV infection by si-RNA transfection. The efficiency of knocking down ERGAL was tested by RT-qPCR after transfecting siRNA 24 h then infecting with MOI = 10 at 24 hpi. Values were means ± SD (*n* = 4). **(E)** The expression of ERG was correspondingly reduced in line with knockdown of ERGAL after infection. The expression of ERG in HUVECs was detected by RT-qPCR after transfecting 24 h then infecting with MOI = 10 at 24 hpi. Values were means ± SD (*n* = 4). *P < 0.05; **P < 0.01 vs. si-NC groups (negative siRNA control groups) and NC groups (infected control groups). **(F)** The protein level of ERG was decreased with knockdown of ERGAL. The protein level of ERG in DENV-infected HUVECs was evaluated by western blotting. ***P < 0.001; ****P < 0.0001. The experiments were performed independently at least three times with similar results.

As we mentioned above, lncRNA-ERGAL was located within 20-kb downstream of the *ERG* transcription factor; thus, we hypothesized that this lncRNA could be related to the expression of *ERG*. First, as shown in Figure 2A, the expression of *ERG* in HUVECs was found to increase during DENV infection. Then, the expression pattern of *ERG* was measured throughout infection and its expression level was found to be similar to that of lncRNA-ERGAL. *ERG* expression also occurred in a time-dependent manner rather than in a virus dose-dependent manner (Figure 2B). Since *ERG* was located near the lncRNA and presented a similar expression pattern, we concluded that there could be a certain degree of correlation between them. Pearson's correlation analysis between ERGAL and *ERG* demonstrated ERGAL expression to be strongly correlated with that of *ERG* (Figure 2C, *P* < 0.0001, *R*^2^ = 0.715).

We used the infectious model (24 h post-infection with MOI = 10) to conduct the subsequent functional experiments due to simulating the early hyperviremia in humans infected with DENV (Figure 1A). RNA interference (RNAi) was used to knock down the expression of ERGAL in order to investigate its functional involvement in DENV infection. The efficiency of RNAi for silencing ERGAL was tested first, and short interfering RNA (siRNA) was found to effectively suppress the expression of ERGAL in infected HUVECs compared to that in control cells without siRNA transfection (Figure 2D). Additionally, the gene and protein expression of *ERG* were also repressed when ERGAL was silenced (Figures 2E,F), indicating once again the association of ERGAL with *ERG* expression.

### Knocking Down lncRNA-ERGAL Impaired Cell–Cell Junctions by Inhibiting the Expression of Junctional-Related Proteins

**Figure 3:**
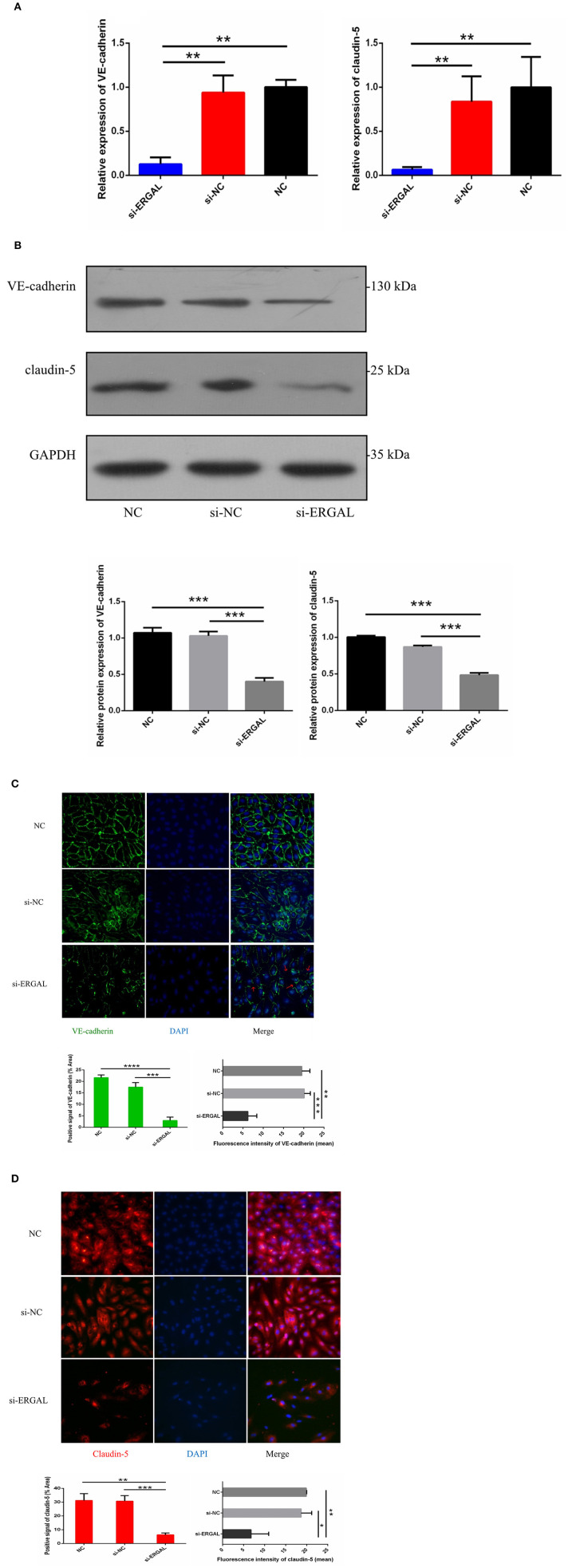
Knockdown of lncRNA-ERGAL impaired the adherens junction and tight junction of HUVECs infected with DENV. **(A)** The gene expression of VE-cadherin and claudin-5 was reduced after DENV infection (MOI = 10, 24 hpi) with knockdown of lncRNA-ERGAL. Relative mRNA expression of VE-cadherin and Claudin-5 was detected in HUVECs by RT-qPCR with knockdown of lncRNA-ERGAL. Values were means ± SD (*n* = 3). **(B)** The protein level of VE-cadherin and claudin-5 was inhibited after infection (MOI = 10, 24 hpi) with knockdown of lncRNA-ERGAL. Relative protein of VE-cadherin and claudin-5 were measured by western blotting. **(C,D)** The distribution and expression of VE-cadherin and claudin-5 decreased after infection with knockdown of lncRNA-ERGAL. Immunofluorescence analysis of VE-cadherin or claudin-5 in HUVECs. Nuclei were stained blue (DAPI), and VE-cadherin or claudin-5 were stained green or red, respectively. Positive signal represented the area of protein distribution, and the mean fluorescence intensity (Integrated density/Area) represented Semi-quantitative protein expression. *P < 0.05; **P < 0.01; ***P < 0.001; ****P < 0.0001 vs. siNC groups or NC groups. Original magnification: 40x. The experiments were performed independently at least three times with similar results.

Because both VE-cadherin and claudin-5 were key junction-related proteins that play vital roles in regulating permeability of the endothelial barrier, we further explored the effects of ERGAL on the adherent junctions and tight junctions between cells. qRT-PCR assay was used to measure the gene expression level of VE-cadherin and claudin-5. Results demonstrated that both of them were repressed significantly during DENV infection after lncRNA-ERGAL was knocked down, compared to that in the si-NC and NC groups (Figure 3A). As shown in Figure 3B, silencing ERGAL also inhibited the protein expression of VE-cadherin and claudin-5 during DENV infection. Additionally, Immunofluorescence analysis demonstrated a comparatively continuous distribution of VE-cadherin along the cell border of HUVECs in both the NC and si-NC groups, whereas its distribution was obviously discontinuous in the si-ERGAL group (Figure 3C). Moreover, a mass reduction was observed, along with more severe fractures among the cell–cell junctions in the silenced lncRNA-ERGAL group, as shown by red arrows (Figure 3C). Analogously, immunofluorescence results revealed the expression of claudin-5 to be remarkably suppressed in the membrane and in cytoplasm in the group with ERGAL knockdown compared to that in the si-NC and NC groups (Figure 3D). Taken together, knockdown of lncRNA-ERGAL aggressively impaired the cell–cell junctions of the vascular endothelial barrier by inhibiting the expression of VE-cadherin and claudin-5 during DENV infection.

### lncRNA-ERGAL Knockdown Promoted Apoptosis of Vascular Endothelial Cells and Increased Monolayer Cell Permeability

**Figure 4:**
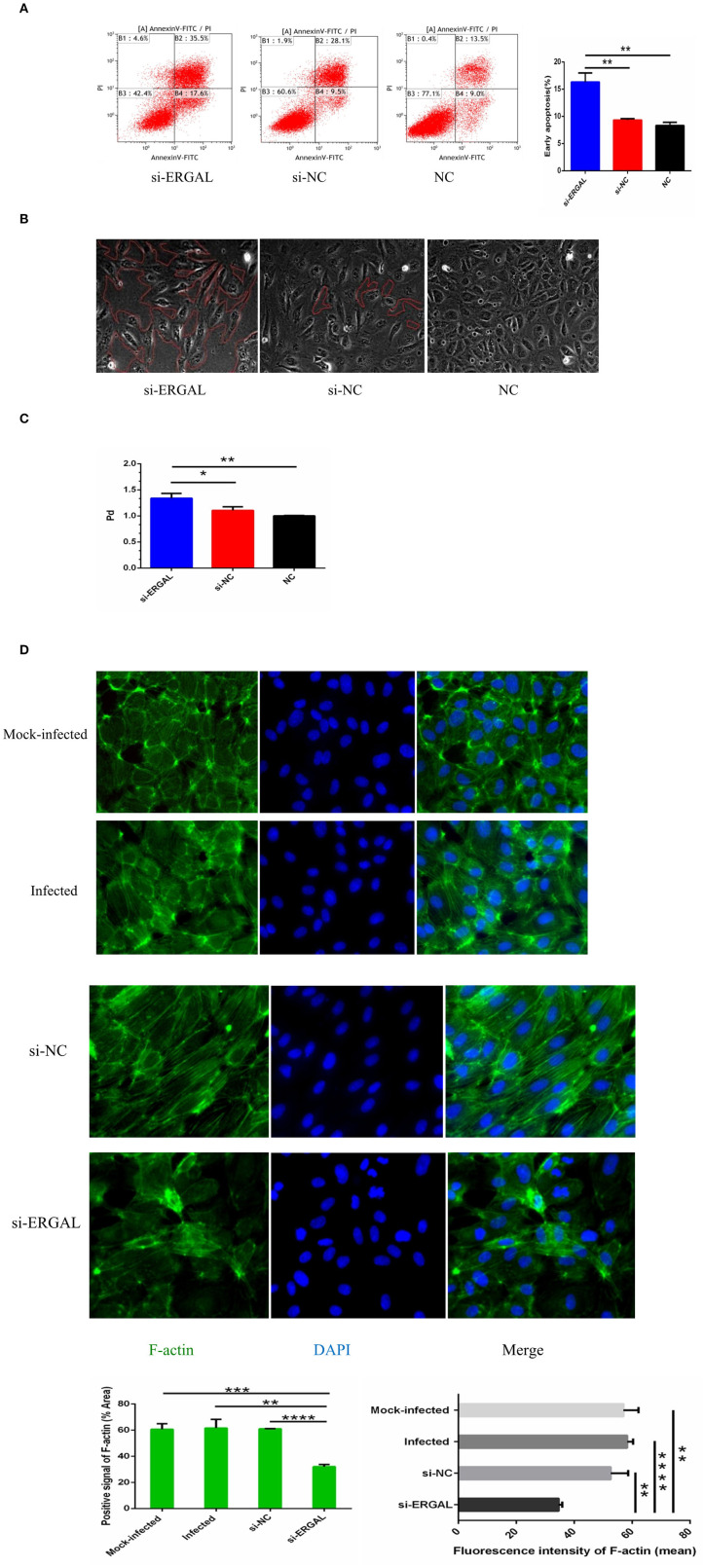
Knockdown of lncRNA-ERGAL promoted vascular endothelial cells apoptosis, increased monolayer cell permeability, and obstructed cytoskeleton remodeling. **(A)** Knockdown of ERGAL enhanced early apoptosis induced by DENV. Apoptotic statues of HUVECs were assessed by flow cytometry. Values were means ± SD (*n* = 3). **(B)** The arrangement of DENV-infected HUVECs with the siRNA-mediated knockdown was sparsely and loosely rearranged, and the gaps among cells were observed to become notably expanded. The gaps among cells were shown under optical microscope (40x), and the gap size was drawn in red. **(C)** The knockdown of ERGAL coincided with increased permeability of HUVECs monolayer to 40 kDa FITC-dextran after DENV infection(MOI = 10, 24 h post-infection). The permeability assays were performed by detected the FITC-dextran permeability coefficient. Values were means ± SD (*n* = 3). **(D)** The distribution and expression of F-actin decreased, and cytoskeleton remodeling disordered after infection with knockdown of ERGAL. Immunofluorescence analysis of F-actin cytoskeleton was performed in HUVECs. Nuclei were stained blue (DAPI), and F-actin were stained green. Positive signal represented the area of protein distribution, and the mean fluorescence intensity (Integrated density/Area) represented Semi-quantitative protein expression. *P < 0.05; **P < 0.01; ***P < 0.001; ****P < 0.0001 vs. siNC groups or NC groups. Original magnification: 40x. The experiments were performed independently at least three times with similar results.

As shown in Figure 4A, flow cytometry analysis with annexin V-FITC/PI fluorescence staining was used to detect early and late apoptosis. The finding demonstrated that the suppression of ERGAL significantly enhanced cell apoptosis, especially promoting early apoptosis (*P* < 0.01), thus indicating ERGAL as possibly an indispensable element for repressing the endothelial cell death caused by DENV infection. Furthermore, DENV-infected HUVECs with knockdown of ERGAL were sparsely and loosely rearranged, the gaps among these cells being notably expanded compared to those in the control groups (Figure 4B). Transwell permeability assays were performed to measure the permeability of a HUVEC monolayer. Results demonstrated the permeability coefficient of dextran (P_d_) of the si-ERGAL group to be higher than that of the other control groups, representing comparatively elevated permeability after silencing ERGAL in the monolayer endothelial cells. These findings suggested that the knockdown of ERGAL aggravated the breakage of vascular endothelial cell barrier during DENV infection (Figure 4C).

In HUVECs that were not infected, cytoskeletal F-actin was clearly structured and neatly arranged (Figure 4D: mock-infected group). After DENV infection, F-actin fibers became disassembled and dispersed drastically throughout the cytoplasm, as well as re-polymerizing into stress fibers along the cell edge (Figure 4D: infected group and si-NC group), indicating that DENV infection could induce the reorganization of F-actin in the cytoskeleton. When ERGAL was repressed by siRNA, F-actin was almost completely depolymerized and less dispersed in the cytoplasm. The original cytoskeletal structure was also severely disrupted (Figure 4D: si-ERGAL group). This finding implied that ERGAL was necessary for F-actin cytoskeleton remodeling during DENV infection.

### MiR-183-5p Regulates the Vascular Endothelial Barrier via Junction-Associated Proteins and the Cytoskeleton

**Figure 5:**
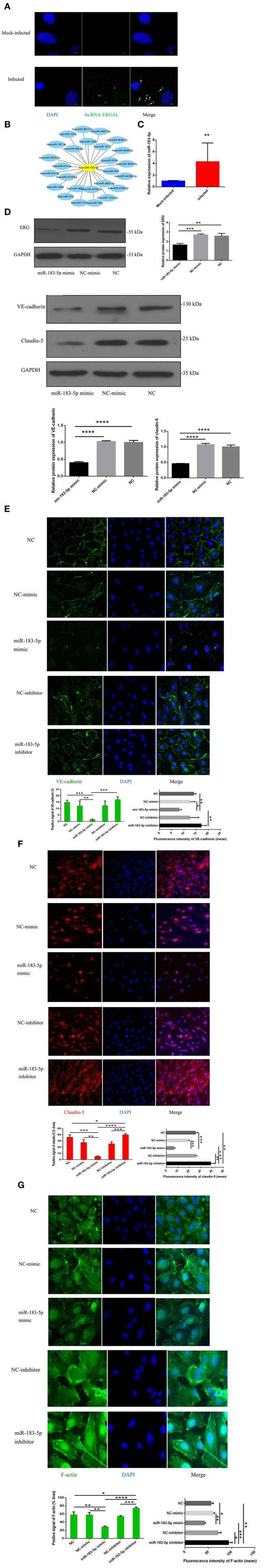
miR-183-5p involved in functioning the endothelial barrier via regulating junction associated proteins and cytoskeleton. **(A)** ERGAL was mainly expressed in the cytoplasm. Localization of ERGAL was detected by FISH in HUVECs. Nuclei were stain blue (DAPI) and ERGAL were stained green (white arrow). Scale bars: 5 μm. **(B)** ERGAL sequence contained multiple miRNAs binding site. The putative microRNAs binding ERGAL were predicted on RegRNA_20_ (http://regrna2.mbc.nctu.edu.tw/). **(C)** The expression of miR-183-5p was significantly increased after DENV infection. The expression levels of miR-183-5p were detected in HUVECs by RT-qPCR (MOI = 10, 24 hpi). Values were means ± SD (*n* = 6). **(D)** The protein level of VE-cadherin and claudin-5 was inhibited in HUVECs with miR-183-5p overexpression and DENV infection. The protein level of VE-cadherin and claudin-5 was measured by western blotting. **(E)** The protein level of ERG was decreased with overexpression of miR-l 83-5p. The protein level of ERG in DENV-infected HUVECs was evaluated by western blotting. **(F, G)** MiR-183-5p regulated the expression of VE-cadherin and claudin-5, F-actin, and cytoskeletal remodeling after DENV infection. Immunofluorescence analysis of VE-cadherin, claudin-5 in DENV-infected HUVECs transfected with microRNA mimic or inhibitor. Nuclei were stained blue (DAPI), VE-cadherin, and F-actin were stained green, claudin-5 were stained red and F-actin were stained green. Positive signal represented the area of protein distribution, and the mean fluorescence intensity (Integrated density/Area) represented Semi-quantitative protein expression. *P < 0.05; **P < 0.01; ***P < 0.001; ****P < 0.0001. Original magnification: 40x.

In order to explore the potential regulatory mechanism, the location of ERGAL was confirmed first by RNA FISH, revealing it to be mainly expressed in the cytoplasm (Figure 5A). RegRNA_2.0_ (Chang et al., 2013) was further utilized to predict the ERGAL sequences, showing that it contained multiple miRNA binding sites (Figure 5B). The above predictions suggested that ERGAL possibly acted as a miRNA sponge, regulating miRNA availability for binding the target mRNA (Tay et al., 2014). While we noted miR-183-5p to be widely reported as an important regulator for many diseases, including cancer and infection, no such report is available to date for DENV infection. We first measured the expression of miR-183-5p in DENV-infected HUVECs and observed that it was highly elevated (over 4-fold) than in mock-infected cells (Figure 5C), which implied its involvement in regulating DENV infection. Based on the aberrant expression of miR-183-5p and its predicted relationship with ERGAL, we assumed that miR-183-5p might be possibly involved in the regulation of endothelial barrier during DENV infection. As shown in Figure 5D, overexpression of miR-183-5p significantly reduced the protein levels of ERG, VE-cadherin, and claudin-5 in DENV-infected HUVECs. Furthermore, we performed a fluorescence assay to visually assess the effects of miR-183-5p on cell junctions and the cytoskeleton. As shown in Figure 5E, VE-cadherin was discontinuously distributed and mostly degraded at the cell–cell junction in the group overexpressing miR-183-5p, whereas it had a comparatively continuous distribution along the cell border when miR-183-5p was inhibited. Similarly, overexpression of miR-183-5p suppressed the expression and distribution of claudin-5 remarkably, whereas knockdown of miR-183-5p increased the abundance of claudin-5 (Figure 5F). In addition, F-actin in the cytoskeleton was depolymerized and degraded in the miR-183-5p mimic group, whereas the filaments were well-built and cytoskeleton was remodeled in the miR-183-5p inhibitor group (Figure 5G). Consistent with our hypothesis, these results indicated miR-183-5p to be involved in the endothelial barrier by regulating the expression of VE-cadherin and claudin-5, as well as remodeling of F-actin cytoskeleton in DENV infection.

### LncRNA-ERGAL Interacts With miR-183-5p to Regulate ERG

**Figure 6:**
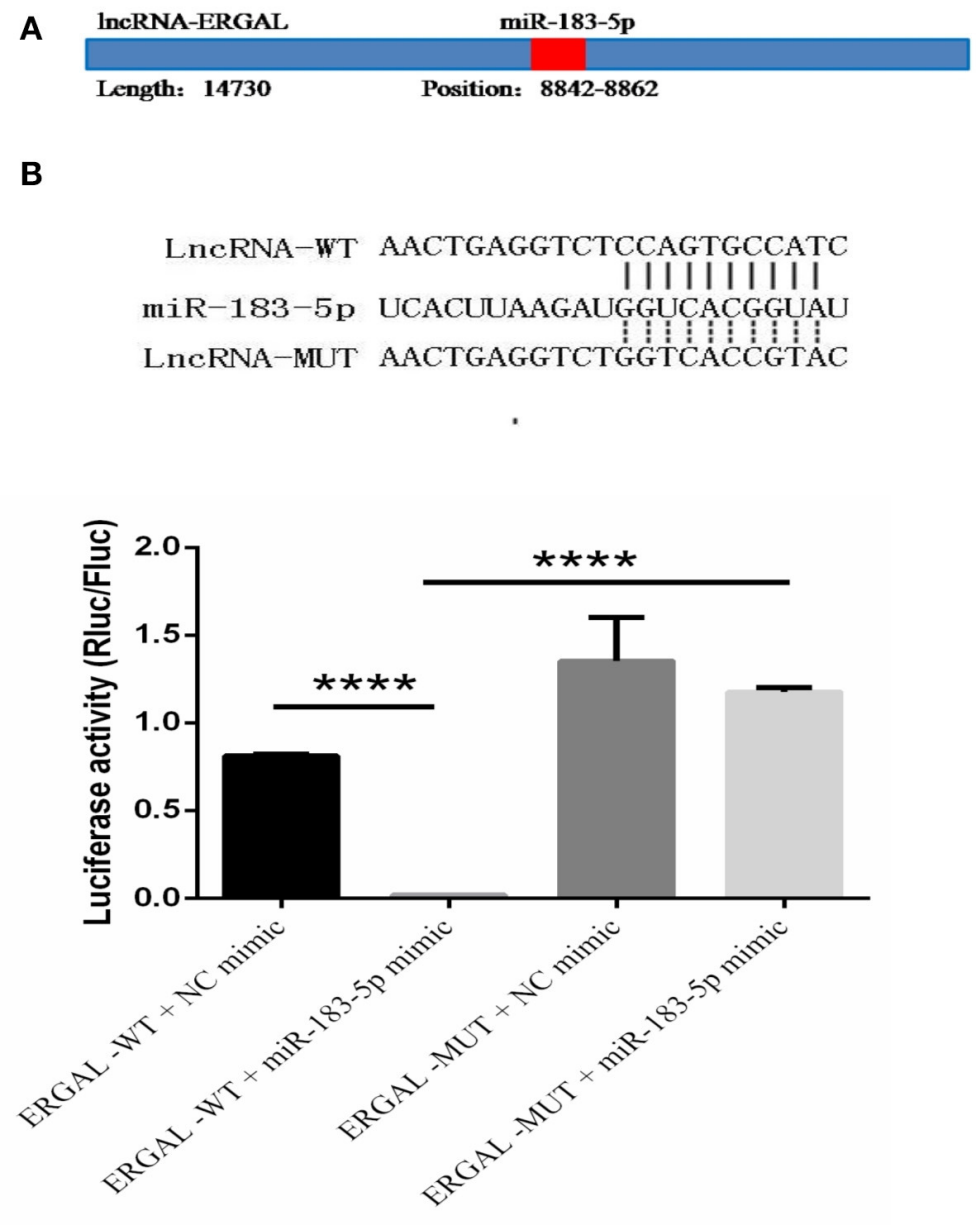
LncRNA-ERGAL interacted with miR-183-5p to regulate the vascular barrier. **(A)** ERGAL was predicted to bind to miR-183-5p from 8842 to 8862 sequence site. The panel showed schematic representation of the predicted binding site for miR-l 83-5p in ERGAL. **(B)** LncRNA could act as miRNA sponge by binding with miR-183-5p. Upper panel presents the alignment or mutation of potential miR-183-5p binding sites in ERGAL transcript. Lower panel showed the interaction between lncRNA-ERGAL and miR-183-5p was proven by luciferase assay. Values were means ± SD (*n* = 3). ****P < 0.0001.

The specific binding sites of ERGAL and miR-183-5p were obtained by bioinformatics prediction (Figure 6A). A dual-luciferase reporter gene assay was used to verify their binding relationship. As shown in Figure 6B, overexpression of miR-183-5p remarkably decreased the luciferase activity of ERGAL-WT vector (*P* < 0.0001), though not of the empty vector. Mutation of the ERGAL pairing sequence in miR-183-5p abolished the interactions between ERGAL and miR-183-5p, and consequently, miR-183-5p overexpression failed to reduce the luciferase activity of ERGAL-MUT vector, thereby indicating ERGAL to function as a sponge for miR-183-5p. The finding suggested that ERGAL/miR-183-5p, as a combination, plays an important involvement in regulating the permeability of vascular endothelial barrier during DENV infection.

## Discussion

Dengue has been a major public health problem for years and threatens almost every tropical country, with heavy casualties and economic loss during each epidemic. Severe dengue is about the development of the severe dengue complications, such as DHF/DSS, and has a high mortality rate; it frequently occurs in secondary heterotypic infection or infant infection. Due to the complex pathology and heterotypic cross, severe dengue poses a special challenge to the development of an effective and affordable vaccine. Thus, there is an urgent need to develop novel efficient therapeutic strategies and exploit new kinds of molecular vaccines against the four serotypes simultaneously. Based on a better understanding of the underlying pathogenic mechanisms, it has thus far remained unclear. Wang et al. (2017) reported that the differentially expressed lncRNAs induced by DENV-2 infection in hepatic cells revealed that lncRNA may serve as a new diagnostic marker and therapeutic target for DENV-induced liver damage. Additionally, the expression of lncRNA-NEAT1 was reduced in peripheral blood of patients with severe dengue, which is related to the phenotype of severe dengue fever, suggesting that it would be helpful to understand the progress of DENV-induced diseases by monitoring the expression of NEAT1 and IFI27 in peripheral blood (Pandey et al., 2017). Therefore, the study of lncRNA contributed to dig out its pathogenic mechanism and explore therapeutic targets in severe dengue.

The distinguishing feature of severe dengue is vascular leakage that implies damage to the vascular endothelium, which constitutes the major permeability barrier for the vessel wall (Ramirez et al., 1984). Owing to the many important characteristics that resemble the human microvascular endothelium, a modified HUVEC monolayer that is suitable for studies on dengue hemorrhagic fever was used as a vascular endothelial barrier model (Jacobs and Levin, 2002). On the other hand, HUVECs can be infected with DENV because the results of immunofluorescence assay showed that DENV antigens were distributed in the nucleus and cytoplasm in the infected HUVECs (Supplementary Figure 1). More importantly, the viral load in cells was measured with different MOIs (MOI = 0.1, 1, 5, and 10) at different time periods (24 h, 48 h, and 72 h) in order to optimize the infectious model based on the fact that early hyperviremia in patients with dengue fever is related to the development of severe dengue (Vaughn et al., 2000). The results showed that the higher viral load was tested in the cells with the larger MOI and the longer infectious time (Figure 1A). HUVECs were likely to develop growth inhibition within 48–72 h, leading to aging and decline and reducing cell viability. Besides, we also found that the virus load in cells was not significantly different between 24 and 48 h with MOI = 10. Therefore, HUVECs with MOI = 10 at 24 h post-infection were selected as the infection model for all the following experiments. Not only could it simulate the early hyperviremia in cells of DENV-infected patients, it also relatively maintained cell viability. We infected this model with DENV (MOI = 10, 24 h post-infection) for high-throughput RNA sequencing and found that the infection could significantly induce a mass of differentially expressed lncRNAs. Furthermore, the differentially expressed lncRNAs and the predicted corresponding target genes were mainly involved in important biological processes closely related to severe dengue, indicating that these lncRNAs might regulate the pathology of severe dengue (Zheng et al., 2019).

In the present study, we discovered a DENV-specific-induced lncRNA (non-code transcript ID: NONHSAT190967, non-code gene ID: NONHSAG082924) associated with the function of the vascular endothelial barrier. This lncRNA, named *ERG*-associated lncRNA (ERGAL), increased 2-fold in DENV-infected HUVECs in a time-dependent manner instead of a virus dose-dependent manner when compared with mock-infected cells, indicating that it involved during DENV infection. ERGAL was identified as a full-length 14-kb untranslated RNA molecule and was transcribed from chromosome 21, with no predicted protein-coding potential; further, it was found to locate 20 kb downstream of the transcriptional factor *ERG*. We further noticed that ERGAL was silenced without DENV infection, suggesting that it might be activated by DENV as a virus-specific lncRNA. Due to the long length of ERGAL, it was currently difficult to construct a suitable overexpression vector; hence, short interfering RNA (siRNA) was used for knocking down ERGAL to detect its functions. Notably, ERGAL knockdown suppressed both the gene and protein expression level of *ERG*. Previous reports indicated that *ERG* is highly expressed in the endothelium and is essential for endothelial cell homeostasis and angiogenesis (Birdsey et al., 2008, 2015; McLaughlin et al., 2010; Shah et al., 2017), as well as regulating junction stability through transcriptional activation of genes encoding junctional proteins, whereas its loss could increase endothelial permeability (Yuan et al., 2009). Our study assessed and showed the strong correlation between *ERG* and ERGAL, suggesting that ERGAL might play a potentially important role in regulating the vascular endothelial barrier function via *ERG* during DENV infection.

Studies had shown that high DENV load infection altered the junctional integrity of brain and lung microvascular endothelial cell (MEC) lines during early infection (24 h post-infection), with the differential expression of adherent proteins, junctional proteins, gap proteins, and adhesive molecules, of which VE-cadherin was up-regulated within the first few hours of infection with a high MOI of DENV, while the expression slowly decreased later (Soe et al., 2017). This might be a protective mechanism against the DENV infection during early infection, possibly by increasing anchorage of VE-cadherin to the actin cytoskeleton, as well as by up-regulating the expression of tight junction proteins (Taddei et al., 2008). However, ERGAL silencing dramatically down-regulated the gene and protein expression of VE-cadherin and claudin-5, leading to intercellular gap formation, as well as disruptions in the cytoskeletal structure, all of which resulted in the imperfection and instability of vascular endothelial barrier during early infection. Moreover, ERGAL knockdown greatly promoted early apoptosis of endothelial cells, thus aggravating endothelial injury leading to damage of the blood vessel lining. In our sequencing analysis, we had predicted that multiple lncRNAs could regulate the XAF1 gene (NM_199139, up-regulated 5.6-fold in the sequencing analysis), which contributed to induce apoptosis in vascular endothelial cells in DENV-infected HUVECs (Long et al., 2013); thus, we believed that apoptosis of vascular endothelial cells were more likely to be related to the regulation of multiple lncRNAs during DENV infection. There was no doubt that apoptosis could cause the decreased expression of VE-cadherin and claudin-5 to some extent. However, as shown in immunofluorescence assays (Figures 3C,D), VE-cadherin was obviously discontinuous and a mass reduction was observed, along with severe fractures among the cell–cell junctions in the silenced ERGAL groups. Similarly, the expression of claudin-5 was remarkably suppressed in the membrane and cytoplasm in the group with ERGAL knockdown, so we assumed that the reduced expressions of VE-cadherin and claudin-5 were mainly caused by ERGAL knockdown rather than apoptosis. Furthermore, previous reports have revealed that *ERG* and VE-cadherin were associated with apoptosis resistance and antiapoptotic signals related to network stability (Birdsey et al., 2008). Thus, we considered that ERGAL might play an essential role in cell survival via regulating *ERG* and VE-cadherin. Taken together, ERGAL contributed to promote the stability and integrity of the vascular endothelial barrier during DENV infection by regulating *ERG*, junctional proteins, cytoskeletal remodeling, and cell survival.

**Figure 7:**
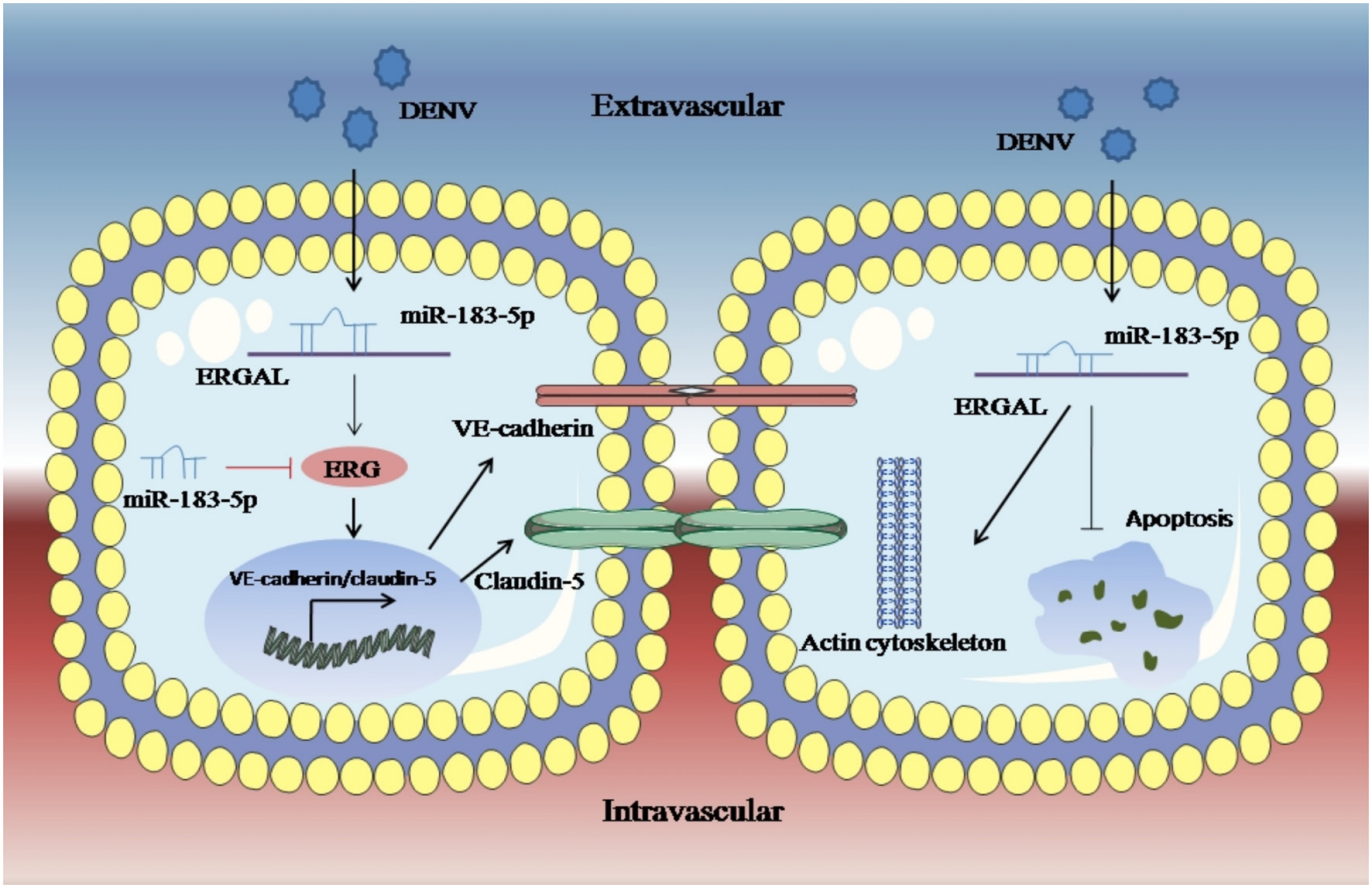
LncRNA-ERGAL was induced by DENV and involved in regulating vascular endothelial barrier during early infection. Dengue virus can activated ERGAL, which could bind with miR-183-5p to attenuate its inhibition on expression of ERG, VE-cadherin, and claudin-5, besides ERGAL could reduce the early apoptosis and promote cytoskeleton remodeling for promoting the stability and integrity of endothelial barrier against DENV challenge.

An RNA FISH assay was performed and found that ERGAL was mainly expressed in cytoplasm, indicating that ERGAL might participate in the post-transcriptional regulation. Furthermore, bioinformatics prediction showed that the ERGAL sequence contained multiple microRNA biding sites, suggesting that it could act as miRNA sponge to interfere with miRNA pathways. Notably, miR-183-5p, which belongs to the miR-183 family, is involved in tumor progression by acting as an oncogene, tumor suppressor, and a biomarker (Rizos et al., 2015; Cheng et al., 2016; He et al., 2018; Meng and Zhang, 2019), and is closely associated with the development of cancer. Additionally, the miR-183 family has been revealed to target a diverse set of host mRNA, some of which have potential effects on viral replication by changing the behavior or resilience to stress in the infected cells. It was also reported that the miR-183 family plays roles in the pathogenicity of viral infection like Epstein–Barr virus (EBV) or human cytomegalovirus (HCMV) (Stark et al., 2012; Dambal et al., 2015; Oussaief et al., 2015), but its role in DENV infection has been unknown thus far. Therefore, miR-183-5p attracted our interest and attention. In our study, miR-183-5p was dramatically up-regulated after DENV infection. Furthermore, its overexpression damaged the endothelial barrier by suppressing *ERG*, VE-cadherin, and claudin-5, as well as destructing the cytoskeleton during DENV infection, whereas inhibition of miR-183-5p presented the opposite effects. As ERGAL and miR-183-5p showed opposite functions with regard to the vascular endothelial barrier during DENV infection, we conducted a dual luciferase reporter gene assay and confirmed that ERGAL could target and bind miR-183-5p, thereby acting as an miRNA sponge to regulate the permeability of vascular barrier during DENV infection (Figure 7). However, there were also some limitations of our study. We used mock-infected cells that were incubated with culture supernatants as the control group without infection when detecting the expression of some genes after DENV infection. Since we could not detect the expression of ERGAL in mock-infected cells, only the infected cells were used as the control groups including NC groups (normal control groups with infection) and si-NC groups (negative siRNA control groups) for functional verification of ERGAL. We also use the NC-mimic groups (negative microRNA overexpression control groups) and NC-inhibitor groups (negative microRNA inhibition control groups) as control groups when detecting the function of miR-183-5p after its overexpression and inhibition. In order to simulate the early hyperviremia in cells of patients and maintain cell viability, only one time point (24 h post-infection) with MOI = 10 was used for all the experiments rather than multiple time points. The possible role of ERGAL in natural infection in human patients was also necessary to be further explored through subsequent animal experiments. Though ERGAL was found to be induced by DENV and regulate the vascular permeability during early DENV infection *in vitro*, it is also very essential to verify ERGAL functions *in vivo*.

Overall, based on what we found in the present study, ERGAL was induced by DENV and promoted the stability and integrity of endothelial barrier during 24 h infection by binding with miR-183-5p, which could further reveal ERGAL/miR-183-5p as regulators involved in the molecular mechanism of pathogenicity, as well as might provide a foundation for a promising target for the clinical treatment of severe dengue.

## Data Availability Statement

The raw data supporting the conclusions of this article will be made available by the authors, without undue reservation.

## Author Contributions

LJ, JZ, and ZJ conceived the study. HW, JZ, HY, and DF provided instructions during the experiment. BZ, GC, LS, and QG designed and performed the experiment. BZ wrote the manuscript. JZ and HW revised the manuscript. All authors contributed to the article and approved the submitted version.

## Conflict of Interest

The authors declare that the research was conducted in the absence of any commercial or financial relationships that could be construed as a potential conflict of interest.
