# Supplementary material for: ERG-Associated lncRNA (ERGAL) Promotes the Stability and Integrity of Vascular Endothelial Barrier During Dengue Viral Infection via Interaction With miR-183-5p
Source: Front Cell Infect Microbiol. 2020 Sep 8;10:477. doi: 10.3389/fcimb.2020.00477 (PMC7506072; doi:10.3389/fcimb.2020.00477)
Supplement: Supplementary file 1 [file Table_1.DOCX]

**Table S1** | The general information of lncRNA-ERGAL.

| **Noncode transcript ID** | **Noncode gene ID** | **Chromosome** | **Start site** | **End site** |
| --- | --- | --- | --- | --- |
| [NONHSAT190967](http://www.noncode.org/cgi-bin/hgTracks?position=chr21:38350109-38364839&nonLnc=full&hgFind.matches=NONHSAT190967.1&PhyloNONCODEGene=hide&PhyloNONCODELncRNA=hide&db=hg38) | [NONHSAG082924](http://www.noncode.org/show_gene.php?id=NONHSAG082924&version=2&utd=1) | Chr 21 | 38350109 | 38364839 |
| **Sequence:** caATAAGTGTTGCACATCTGCAAAGTGCCCGGCACTGGGCTAGGCTCTGGGCATTTCAAGATGAATAAGACCCCACTTCTGAATGACGCAAAGAGCTTTCCAGCTACACAGTGGCATTTGTAATGTCACAGGCAGTCCAAGGAGGGAGTGGTTACTTCACAGAAGTCACTAGAGAAAAAGAGTATTTATGCAGGGCCTACAAGGATGGGAAAGATTTTGGCAGGGAGAAGCAGAGGGCTAAGGAGCTCTAGAAAGATGAGTTTCCAAGTATAAGCAAACTGAGGAAGCTGGAAATGAGGAAAATGGGGTGGTCAGCATGGCTGAAGCTTGGGAAATGCGGAGCGGAGTGGTGGCAAGTAAGCCACTGACAAATTGCCGAGTTTCTTCAGAAGTAACGAGTGGGGCTTTTCTCTGTAGTAGCTGGGAACCAAAGAAAATGTGTGCGTGGAGGCAGAGGCAGTCAGGGATCTGATCGGAAAGTTCAGTGTGCTGTCAGGTGAGGAATGCATTACACAGGTGGGTTGGAAATACTGGGATAGGAGGCAAGAGATACAGACTTGAGCAAATGCCTGAGAGGGAAGCAGTAGGTTGCTGAGCAGGGCATTGCCGAGGGCAAGAGGAGAAGGAGGTGGCTGTGCAGAGGGGGCAGGCCACCCATCTGGACTCTGTCCTGGCCCCACTCTTGGCAGCTATGGGACCAGGTTTAAGTGGCCTCCAGTTCCTCTGGAGAGAGAGAAGGACTGGGTACAGCAGCCCCTCCCCCAAGCCTATTGAGACATCTGTAGGGAAGAGAGTTCAGCTCACTGGGAAGGAGAAGCCTTCAAGGCTGGAGAGCGCTGTGGTGATGTGGTTATCTAACTTTGCTGTGGGTCAGAATTGCCTGGGCAGCATAGCAAGCGGTCCCATCCCTGGAAACTCAGACTGGGCTGCCTTTGTAGCTGCACCACACTTTGGAAAACCCTAATCTCACATCTGGGGTGTTTTCTGATTCTCTGTTTGGAGGATTCTGAAGCTCAGAGGGGCTGGGTGGGTCCAGCCTGAAAGGGGCACATCCCATCACCTCTCTCTCCTCCACCATTCACAGGGACGCCCAACACGATGCCCTCCGTGTCTTCATctctccctctccacctcctcagtccctttccctGGACTTCATGGGAGCCTGTTGAGCTCAAAAGATCCCCAAGAGCCAAGATGCAAGCCTGCAGATTTCAGGACAGCTGGTCCTGGCGCTGCTGGCTGCTCGCAAGGGCCGGGCCTCCTCATTGCCGTCACCAGCCCCCGTTGGGCCTCATCAGAGCAAGACCAGGCTCGGCTGATGCTCGGCGCTGTATTCCTTGACGCCTCCATGAGGCTTGCTGCCcagcatgtcccaggcttgctggaaatgctgaagctcaggctcacctcagacccacgactcagagtctgtatcttttccagatcctggagtgatcgcatgtgcacgtataaatcggagaagcTTTGGCTTCCATAGCCTCTGCTGGCTGTAACTAATCCTTCCTCACCCCATACTGAGGGAAAAGAAAgaagggaagggagggaaaggaagggaagagaaaggagaaggaaggacagggggagagagagagagagaaagggaaggaaggaaggcaggaaaggaaagaaggaaggaaggaagggaggaagggagggaaggagaaagagaaaggaaggaagaacaggaggttaggagagaggaatgaaagaaagagaatgggagagggacagagggagggagagagAATGATTCTGTGAGTCTCTTAACTCTGGTCCTCCTGTTACACATCTTTTCTTCTACTTTTCCTTTGTTTCACACTTAAGCTTTGAAGATGTAAAGTAGGACCAGAGGGTGAGAAATTCAAGAACCTTGAAATAAAAGACCTTTCTGTTTGCTTTTCATTTCCTAAAAAGGAAGTCCTCACTGACTGTTCCTGTAAGGGATATTAAGCTGTGTGGATTGTGAGTGATTCGTCACACAGCAATTTGGAGCAGGAAGCACACGAAACCCTAGAGCTAGCCTGCACGCAACTAATTGAAGCCCTCAATTAATCGGATTGGCTTCCACCTTCAGGAGAAAAGCAAATACTAAGAAAACAAATTAGCACCTGGAATTTAACAACTTCCCTTTTTTGGTGTGGGGGCGCATTATATTCAGAGTACATGATAACTGATGGCTCGTCTCATAGGCACAGACATCTTTACAATCCTCTAAggcagagtggtgtggtggaaacggagttggcagaggcatcagatctgcccgggtttccatcccagctccagcatgcatgggccatgtcgcttggcaagcagtctcaccactcagagccttgatttcaggattaaaaaaataataacctattcaacagaatcatggtcgagaagattaGGGGTTTGTGCAACTCCTCACCTGGTGGATACTCAACCGGAATGAGTTCTCTTTCTCAGTTCCTGCCTACAGATCTATAGACACTTAGCTTTAAAAGCTTTGATTATGGCCAGTTGCCTGGTCAACCTAATACCAGCAGAGGAGTGTATTTTCCCTACTATGGTGGAACAGAGGTAAAGGTTTTCCAAGTTTGAAGCAATCATACAAACAAAATAGGAGCCCAAAGACTTCTGCGGAGCTTTGGAATGAGAGATGCCCATTCTGGTTTCAGAAACGCCAGCGCCATCTCTTGGAGGAGCATGAAATTGTTTCCTTGGGCTGAAGAAGCATCCATGGGGCCCCAGGTAGGTTCAGCTGGTCCTCTCCTTCTTTCAACTGTAGTTTCAGACTGGAACCCCAGGGCTTCTTCCAAGGGAAACATTCTTACTTATTTCCTTATAAAGGTCCTGCATACCTTTGTTTGTCTCTCTTCCAATCAAACCATTCTTTCTCTCTAATTTCATCGGGTCCAGTATTTCCAAATTTCAGTCCCAGTTTGTAAATCTACAACTCAAGCCAAACCTTCAAAGACTGAATTTTCTAAGTGGTTAGTTATCCTTCTAACACAGAGGGTCTTCCTTCTAACACCCCAGCAGAGGCCGGGtagcctagcactcaagatcctggactctgaagctagaccactgtgtttagcatcccctcagccttccaactgtgtgaccttggaaagttacttgacctctctgtgcctcagtttccccacccacaaaatgaggctgacagcagacaccacctagaatggttgttgcagcattacaggagttcacacctgcagcactcggtgcaggcctgTGACAGATCATGAGCAAGTGTTTGCCATCATCTTGGTTTTCAGTGGCTGTTCCCTTACACACCACTTACGACGCTGGGCTAGGATTTCACTTGACTTGTCCAGGTTACCCACCAGGCGGTGTTTCTGTAGCATGGGCAGTAAAGGCAGCATAAATCTAGGCCACACTCACAAATGCACATTGTTAGCACACAAACCCCGTGATTCAGATGCAGGTGGTCTGGGGGCCTGATTTTTAGAGCTGCTGCACTCAGTTTTGTGCTCCTAGGGGGCAGGGACTAGCTCCATCCATCTCAGACCCCAGCCTGCATGCAGCAAGCACCCGCCCCCTTGAAAGTGCTCAGCTCAAGTTGCTTTCCTTCTCCATCTTCTTTAATAAATGATCTTGGAGTGAACAAATGGGAGGCTGACAGTATTACACTAAAATAACAGGAGTTTCTGGTCACCTGAGAAGCACAATGGTCTCTCATCTTCTCCTTACTCCATTAGGAGTTCATATGCCTGCATTTTCCTCATTAAATCTGAGGTGCTTTTCTGAACTCTATCTGAGAAGGAGCTGAAGTAATCAATGAGTGTCTCACACACAAACACATGGAAACAGGTTCTTAGTGAGAGCGGTGGGGACTCTGACTCCAAATTTAATGATTTTTCACTAAAAAGGAATGACGCTTCCCTTCACTAGAAAGCAATGATGTCTTCGGTGGTTGCATCAATGACCAAACATGTATAATAAACTGCTTCTTTGAACTTGAGGACATCTGATTAAGAGATAGAAAGCAAAACAAGCCTTCACAGATGGTAATAGTGCTGGGACACATCACCTTTTGTGAATAACTGAAGAGCATATATTAATAACAGAAATCACATTGGTGTGCCCTTACTGAGGATCGACAAAGGAGAACACGTCTATGTTTTGTGGAAACACGACTGAAGCCTTCATCCAATGACGTTGGCCGGCCCCGCTCTCCTCGTGCCATCAAGCACTCTACGCTGACTCAGTAGAGCAGCACAGCCCTGTGTGTGGAGAGATTCCCATGCACCATGGGAAGAGGGACCAAGCCTGGGCCCTGGAGGGCGCAGCCGGTGCAGTCTGCAGGAGGCTCTGTGTTTCAATAACTGTGGTGTCCCCACACCCCAAGGGCCATCTGTAGATCTGCTGCTCCAAAGTCTCTCACAGTGGCTCTTCTTTGGAGGTCACTCTCCAGTTAAGTGGGGGACCAACTGCAAACTTCTCCCATCAATATGAACTGCAATATGATATTGCAGcaaacaaaacacacacacacacacacacTCTCCCAGAAGCATTTAACAGGAGAAGTGTGTACCTACACATTTAGTCCCAAGATCTCTTTCCAGGCCCTGTCCAGGTTCGTGGTGGAAGCCTGTCCTCTGCACACTGGCTTCCCCAAATCTTCCCATATTCCAGAGCAGGGGAATCCTTTCTCCATCTTTTGGCTCAGATCTACTGCGGAGTGTGGGAGAAATATTAAAAGTAATGATCCTTGATGGTTAAAGCTGCCTTATCAAGTTCCTGCCCCTGGGTTTAAATAAAGTCTCTTTTTCTGCCAGCTTTAGATAAGGATGGAGGGTGagagaaacagacagagacagaaagagagagagaagaagggagagaggcagagaagcaaggactctatagggaaagaaagaGCTTGGACATTAATTCATTTTATGAATATTTGTTCTACTCTTCTTAAGAACAAGGTTTGTCCCAGGTGCCTTGGGGGCAAGAGAGAATTTAAGGGGTGGCAAGGGGCCACTGTCCCGCATCTTGGGGACGTTGTAGGCAGGCAGGTAGAGATGTGCTAAGGTTCCCGGGTCCTGGAGATGCCAGGCAAGTTGGAAAATGCAGAAGAGGAGGCTGGGGTGGACAGGGGTCATTTTGGCTGAAGCTGGTTATGAGAGGCTTAGAAGGTGAAAGTGTCTGCTGTGCTGGGCCCTGAAATGTAGGTCTTCTTGGCCACAGTGGGAAAGGGGAGCCCTAGAGGTCCTTGAACAGTCTGAGTGACAACACCTGGGCTGCCCAGCCCCAAAGGCCAAAACCAAGACACGTCTTTGTCCCAGAGGTTGGGCTTGGTGGCTGCAGCTGTGGATGCTGACCCTTTCATGGGCATGGGGACAGGTGAAAAGCAGGAGCGAGGCAGGCAGGAGTGGGTGCCAGCACCAGCCCACAGCCATGAGGCCTGTGCCAGGCCAGCCTGTGGGGACACTGGGTCCCCGAGAGAGACATGGAAGCTCCCGCCAGCAGGAGACTTGAAGGTGGTCAGTTCTGAAGTCCTCTCCTCCTTAGGCCTGGACCTGGGGACACAGGCTCCAGCCAGCAGCAGGAGCCCATGAAACTCATTTGTCCCAATCTTCTTCCCAAGTCTCAACAGCTTTCGGGGGGTTCTGTGACAGATGCCCCACTTCCAGCTCCACAGCCAGCCCTACGCTAACTCTGACAGATGAAAGGCCTCAGAATCCCCTTCTTCAATTGTACTGCACCCAACTTGTGGAAAGCAAAACAATATTAAAAATACTTTTTAAGAAACTCTAGAATATATTGTCATCAAAGTGATCACGGTTCTGCTTATTTTTGGTCATCGCAACCAATGTACTGAGCCCCCTTTGCCCAGACCCTAGCAGGTCCTGAGTCTCAGCGTCCACAAAACGAGGCTATGGCCCTTGGGGAGTTCCGTGCGGTGCCACAATTGGCCTCTGAGTTACTCTAGAGTGATAAACGCAGCCTTGGCAGTAAAATACCAGAGAGGTAATGAGTGCGGACTCCGTGCTCCCTCCGCAGGGGCAGGTGCGTGAGGCTCTAGCGCCACCTCGTGGGCCGTGGGATTCGTGCCCATCTCCTCCTGGGCGGGAGTTCCTCTGGAGCCAGGAACTCTTTGGGCTCAGGGTGTCTTTTTAGAAAAGCTTCTGACGTTGGTTCTTCTTTAGTGGACCTTCTCCGGTAAAACGGGGTTAACAACTCCAAACTTTTCCCCACCTAAAGGGATTGAAAGGCTGTTTCAAAGGAATTAGAGCTCTGAGACTGCAacccacaaaacacacaatagcatatacacagagatgcacatagaaacatagacacacagggacacacaccagagatacacacacacacacacacggacaggcagacacagggccacaaacacatacacagagatacacacagaaacatatacacacagggacacacaccagagatacatacacagacacacacggacaggcagacacagggccacaaacacatacacagagatgcacatagaaacatttacacacagggactcacaccagagatacacacacacacggacaggcagacacatggtcacacacacacacacatacatacaGTGATATTCAACAAGGGAAGGGTGCACCCACAGGCTCAGTCTTGAGACCTCTCTCCAGGCTCCTGGGTGGGTTGGGGAAGCTGAGCCTCCCTGCCTGCTCATAGCACACAGCCTCCACAAATCTTGCTCTATTCCAGAGTGAGAAAGGAACGTTAAAACTGATGATCCCTGATGGCCAAGGCTGCCTTATCAAGATCCTGTTCCCGATTCAATAAGAAAAGATGCCTCTTGTGCCAGCAGCAGATAAGGCTGCAGCCCTGGGCGTTCCCAGGAGAGCGAACAATGGAAGCCCCCAGGATGCCTCTATATGAGGTAGGATGGTTACTTGCCCAGTCCCTGCCTGCCAGGCCCTTATCTGGGAGGCGCTGGTTCGGGTTGCACCCTCCTGTGTTCCTAGCGGGGGCTGCTGCACACCAGGCTTCTGAGGCCTTCCTGGTGGGGGTTGGTGGAATGGCGGCGGGGGGAGGGTTTTGACGGTGCATCCTGAAGTGCTCCCTGCACCTTTCCCAGAAAAAGAAAAGGATGCCATGAAAACACTTCACCCTAGACATGGTTTACCCTCGGGAAAGCTGATGTGAAAGAAGTTCCAGGGGGCCAGGGATAGAAAAGGATTTAGAAAGATGAAGGCAACAGACAAACGACACTTACAAAGTGCCCAAAGTCTGCAAGTCCTGATAGTCACAGGGAAGGACATCAGCTGGCGGGAAACCTCACCTTTTaggcctgcatgtcagtcttgcactgctgagctgtgtgaccctaggcaggctacttaaaccctctgtgccctggcatctccatctacagaataaagcttctaacagcataggcctcacacacctgtgtggagaccccataagtcgacaacatgtGTTTACCCGAGGAAATAAATATGGGTGCCCCTTTAGGCCTGTCCCACAGAGTTATACAGCTGAAAGACCTCAGAGTAGGTCCACACCCCTTACCAAGTAATGCAGGCCTTGATTAATTGCCACTTGAAGGGAGGTGGGGAGAAAGCACCCTGTCACATCACAGCTAAATTTACTAAAAAAGAAACTCTTTGCCCACTTATCTTTCGAAAGTGCCTCACAGCAGAGACAGCCCCAACCCCTTCCTCTCTCTCCAGCCTCACCTCTCCTCCTAAAATTCAGTTCCTCCTCACACTCTCAGTGCCTGGCCCCGGCcatgcattagatgccctgaagagcttttgaaaaggaccaatccctgggccctatctcaggtgtgtctgagcatctgtagattttagaagctctgaggaggatttacatGCATTTAGGGTGGAAATCACTTGTAAGTAAATTTTTTTAACTGTTCCCTGTTAAAAAATATGAACCAATGGACCAATGGACCACTTATGGCTTCTGCTAATTCTCTTacatatgcccatggtaccttgttgcagtatcatttgtataataaaaagattggagccaaccagaaagtccatcaatggggggaaaattaaataaaatgtagttcattcagcaatggaatactatgcagctctgaaaaatgaatcaggaagctttctatgaacagatgtggaaagtctccaatgtatagcattaagtggaaaaagcaagacataatggtagtgtgcacgttctatattacaaaaaatgtgaataaaggcagtttatattcatgttttcttggatatacatttaaaaaactctaaaaagatacacaaagaatggtCTAGTAGTCATTATAGGGGGTCTGGGAAGGCAAGAGATAATATATCATATTTTCAAATTTTTGAATTATGAGAACATGTCATTCATTCAAAAATTGAATGTAAAAAGGGTTAAGAAAGAGAATAAAGCAAGGGCCGGGACACATCTCCACTGTCTTCCCATGGCCCTCTCAGAAGTGGATCACACCTGGTGCTAGGCAGGGGGACAGAAATTGCTGTGGCCTGAGCAGAGCCTGGGTCCAGCCAGCATCTACACAGCCCACGTCCTCCCAGGGGAATTTCTCTGTGCTCTGTTTTGAGGGGATGGTCCTGGTACTGGAGTAGCAGAGCCAGGTCCCTGCTTCTTGCATAACCCTGAGGCTGCCCTGTTTGCTCTTCTTGTGTGGAGGGCATTGGCAGAGGCCTCTGGCCTGTAGAACCTTTGTGAGCTGAGCCACAACTCACTTCGAGCTGTTCTCTGCCCTGGCTTTGCTACCCTGAGGTCTCCAGTGCCATCCACACCTTCCGTCTGCCACTGCCAACAGCCACAGGTGATGGATGGGGACCATAATGTGAGAGGAGAATTGTGTACTGGAAATCAGCATGAAGGGATATCCAAAGACCTGGAGAAACCCAAATCACATGGGGATGGGAGAGTGAAGACCCAAGGGAGGGATGTGCGGCAAGACCTGCTCTGAGCTCTGAGGGTGCAGATGTAATCAAGACTGAGTGCTGCCCCACCAGACCTGTAGACGAACTCTCCTTCTGGTGAGGCAGTGAACCTGCAAGCACGGGAAGGTGGTGGTGGGTTGGGTGCCATGGAGGAAATGAGGAAGCAGGAGATACACTGGGGGCCAGGTGGGGTTGCAGGTCTCAGCAGGGTGGGGCTGAGGTGGTGATGggtgagggagtgagctgtggagtatccagacagggaaagcagcaagtgcaaaggtgctgggacaggagtggattttgcgggtctgcagggcagtcggtgtactgggagtggagtggatgaggtaggacatggaggctacagagtctcagaacccgtcagaggctggtgtgcagtcaggttctgcagggcattgttggccactaggaggattacaactttttctcttaccccaagtggacttgaaaccattggagggtctgagggaagagtgatatggtcagatgaagtttttaaatcatgtatttaattattctagttgatgtgtagagaatggaattggtgagggaggagagagtagaagtagggggacaagttaggaCTGATGGGAGATTGAGTTGCTCCTTGGCCGTCACAGGATTCTGCATCCTCTGCCTTTACCATGGGCCTTGCCTGCCTCTCCCAGGGACAGAgtgtacccctccaaccttattgactgcgcatttggccatgtgacttgctttgcctcgagggacatgagcaaaggccacatctgagcagaagttttgagggccattgcctggttggctcttgcttttttccctctgctctgagaagaggcatttccaacagggggtaccctccagtccaggtcctagaatgggaaagcctatggagcagggctgccaccgtggcacacagccatgacagaggtgggagtgagaatgagatggctgtcgtcgtaagccttggagatgtggagagtgctGACCGACACAGGGGCCCTTGGGGTACCAGACTACAGAGGCACTGCTGGAACGGTGTGAGGTGAGTTGACTTTGTAATGTTTTCTGAACAGCAGAGCTACCCAAATTGGAGTTTGATGTATGGCATTAGGGAGAGTTTGAtttggtttagggatatgttaagttcgagatactcattagacatctaggtagaggctggagttcctggaaaatgtcaactacaggtacaacttggagtcatcgtctcacatttgcaaactcaatatatgcaaatggtatgaaaagtcatgaggctatacgtgatcaaccagagagagggtgtagaaagaggtgtcttcttaggacctggggcactccagcatGTGCAGAATGGAGATCTCAAAGGGCGAGTGAGCACTGACCTGGTGGTGGCATAGGCAGGTGCATGTCAGGGAGAGAGAACCTGTGTGCAAATACAAGGAAGGTGAGtgacatggtttggctgtgtccccaccaaaatctcatcttgaattgtagctcccataattcccacatgttgtgggagagggcaggtgggacatacttgaatcatgggggcagttctcgtggtggtgaataagtctcgcgagatctactggttttataaggggaaactccttttgcttggctctcgttctgtctttgctggctgccaggtaagatgtgccttttgccttccatcataattgtgaagcctccccagccacttggaactgtgaatccattaaacctcttttccttttataaattacccagtcttgggtgtctttatcagcagcatgaaaacggactaatacaGTGAGAGAGCATGACACCTTCAAGAAGGGGGCAGAGGACAAACTCTCCAGCCTGCCTGGGCTCATGCCCAGCACTGTGGCCCTTCTCTATCCAGGAGCCTTCCCTGTCATTATGCACACCCTCAGTCACTGTCCTCTGCTTGGCAGCACCTCTCTGGACTCACGGTTTGTGGACCAGTTTGTTCTCATTAATCCCACGACTTAAATTTCTAGCACCCATGACACACAACCTTTCCCTCTCCTAACTCAACCCCTTTGTCACTTTCCCCCACTTCTGACGTAAAGGTAACCCATCTTCCCCAATTACAATTTCTTGTGGCCCAGCCTTGCCCCAGTACCAATTTTTAGGAGATGATTTTTGTCACCATAGGTTTTATTTTACTGTGAAAGGCATAAGATTTGTTTGGGGCTCTTCTCTCTCTATGTCCACCTTCCCTGCCCTCAAGTTCACCATGCTCCCCCAGCATGATCTCCAGTCTTCCCTAATGGCTGTTGGCTCCACTTCCCTCCAGCACTCTAAGAGTTATCAGCCTGCAGAATTATGAGCTGCTCAGGTCACCCTACAGCAAGTCACTCCTCCCACCCAGATCATTCTCCCTCAACCCATCAGAGCAATCCCTGGAAAACAGGAAGGTGGCCCTTCACTCCCTCCCCTGCCACACTCCCGCCTGGTAATTCCACCATGGCTGCTTTCAGCCTCACAGGGACCTCAAGTCCCTGGATACTTCCATCCATGCCATGATGTCTGGAACTCTCAAGAAATATTTTGGACAGTTTATCTGCCTGCTCTTGGATTTATTGAGCAGGGAAGAAACTCTGTAAGTTCACATATCTCTACTCTATCCCTGACAATTTTTTCTGGGACTGGAGAGGAACCAGAAAGGGAATGGAGTTGGGGATTGAGGAAAGAGGGGAGAAACAAGAGAAGGGAATACCTTGGGCAGCTGGGCAAGAAGAAACAAAGCCACACCATCCAGAGACAGGGATTTTGGAATAACTGCAAAGTCACAAAAGTATTGGGAAGATGACTTAATGCCCTTGAATTCAAGTCCTCAGACATACACAGATATTGTGGTGATCATCATAGCAAAGCTAGCCTTCCCAGGAAAGAGAATGCCAGAGGTTTTCCTCAGAGGAGGCATAGAATTAGCCTAGccagtgaccagttagccagtgaccagttaaaggacaagtgTTGACTTTGACTTTACCCCAGTCCACTCACGCTGCCACCCCCATGCCCTACAGACCCAGCCATGTCCTTGTGTAAAGCACACCAGGAGACACCAAAGGTATCACAGAGCAGGTCCAAGTGGGACATGGGTTCTGAAACCTTAATGCAAACCAATGGCATGCTGGTATCTGATCCCACCATGACCTTCATCTCGAGAAAGGTGCCACGAGGCTGTTTCTTGAAAGGAAGTTTTGTTGACCTGGTTCCTATACTGAATATGACCACTCACTACCTGGATTTCTTTAGCCATTTGGAATAGGAGCAAGACCACAAGCGCTCATTACCTTCCTGCATGAAATTACATCTCCTGGGAGGAAGAGAGCCATAAACATCAAGGTGGGTGTCACCTGGTCTTAATTTCTTGGCTTCATTAGAAAAATCTGAGAACTTGGAATGACATACCCACAGAGAACATTTGGGTTTACATAAACTAGTATATCCTTCATGTATAAATACATAGTTTAATGTAAAAGAATTGTGGGCCCAAACCAACAGTTTTAGGGTATTTAATTAGGATATTATTCAATAAGCACAAACGGCTCTTCCTAGGTCGGTATTTGATTCACCACTGAAAAATTTGAAACTAGGAAGCAAATCTCTAGAGAAGTTGCATTTATTATTTTGGAGTAATGGTAAACTTTGGGGATTTGATGAAAACGATGGGCCATCTCATCATAAAAATTCACACATACAAACATTTTTTTCTATGCAATTTAGAGGGTACCCAGCCCTAGCTAAGAATCTCTGATCTATTGATTAAAATTCTAAAATAACTAAAATAATATGCAtttatttatttatttatttattttgagatggagtcttgctctgttgcacaggctggagtgcaatggcacgatcttggctcactgcagcctctgcatcttgggttcaagtgattctcatggctcagcctcccgtgtagctgggattacaagcacgtgccaacacgcccagctaatttttgtatttttagtagagacagggtttcaccatattggccaggctggtcttgaactcctggcctcaagtgatccacccacttcggcctcccaaagttctgggattacaggcatgagccaccatacccggccCCAATATGCATTTTCTTAGTGCCTGAAAGCTACGATGACATGGGCATGAAATGGGCCCAGTGCAGACTACATGTGTGAGAAGAGACTTCCTTCAGGCCAGCACGACActtcttccctatggtcacaagtgcctttttcctctttgtgtaacaactcctctctaaccatctgcagtttgcatgtgttgaaccaaaccctaactccaaaaacttaggtctggtcagttgaaacctcccatttatttggctacagtgatgggtttggggatggatatgtgaccaaagtcagtgagtgagactcaattccaggactctcttgagactactgggaaaaacaatgctatttctgttggagtggttgagaggccatgatgtaagccagggattctcaaccagtggtgatttcacctcccatgggacatttgggaacacctggagacatttttggttgtcacttctagggatgtgctactgacatgtattgggtagaaaccagggatgcagttgaacctcctacaaatgcatgggacaaccatccccaacaaagaattattctgccacaaatgccaataatgcagaggttgagaaacgcttctgtaagcttagagtttgagcaactattttggtgttgcataaaatgagcctgcccaagactgaggccaactgaaaggagataaagctgagagatggaggaagggcaagtcatgaagatgtcctttgagatctgggatgcatctctgtctgaagttgagccctggactttacaAAACTGTATCATTTACAAGTAATTGCTTCCTGATTCAGCCTTCTGTTGATTCAACAACACATGTTCGTTAGGTGGCTGCCAGGTGCCAGGACTCTTACTTTCATTGAGTTATACCAGCAACGATGTTAATGAGAAACAAATAACACAGATAAATAAAGAAAATAGAACTGGGAATGGCGGATTGCATACAGAGGCTAATTCAGATGAGGAGGAAACTGGAAATCAACAACAGAACATCCTCCGTGTGCATATATTGTGGTCTATGATGTATGAAAATGTAAAGTACATGATATTTGGTGCTCCTGAAAGAGTGTGGCTGCAGTCACAGTCATCAAATCCTTGCAGTGTCTTCACAAAATCTCAAGTTCAGAACCCCTCTAGGATTGCTATGTAGGTACTAGTATAGAAGAGAGCATTTCTGATCTGTAAAGAGAAAACATGCAGGATTCTGCCTTCAGATGGCATTTTATTTCCTGGCTGGGGGCATGAGACAGACTTTTGGTATTGAAGGACTTTGACTCCTagatgctggcaaggctgtggagaaatagaaacgcttttacactgttgctgggaatgtaagttaattcaaccactgtggaaggcagtgtggcaatttctcaaggatctagagccagaaataccatttgaccaagcagtcccattactggatatatacccaaaagattataaatcattctgttataaagatgcatgcacacatatgtttattgccacactatttataatagtaaagacttggaaccaacgcaaatacccatcaataatagactggataaagaaaatgtggc | | | | |
