# Supplementary material for: ERG-Associated lncRNA (ERGAL) Promotes the Stability and Integrity of Vascular Endothelial Barrier During Dengue Viral Infection via Interaction With miR-183-5p
Source: Front Cell Infect Microbiol. 2020 Sep 8;10:477. doi: 10.3389/fcimb.2020.00477 (PMC7506072; doi:10.3389/fcimb.2020.00477)
Supplement: Supplementary file 2 [file Table_2.DOCX]

**Table S2** | The primer sequence of lncRNA-ERGAL, ERG, VE-cadherin, claudin-5.

| **Gene Name** | **Forward primer sequence (5`-3`)** | **Reverse primer sequence (5`-3`)** |
| --- | --- | --- |
| lncRNA-ERGAL | ACGCTGGGCTAGGATTTCAC | ATGCTGCCTTTACTGCCCAT |
| ERG | AGCACAATCTCATCCGCTCT | AGTGCTGGCCATAATGCGAT |
| VE-cadherin | GAACCCAAGATGTGGCCTTTAG | GATGTGACAACAGCGAGGTGTAA |
| claudin-5 | CTCTGCTGGTTCGCCAACAT | CACAGACGGGTCGTAAAACTC |
| GAPDH | CGCTGAGTACGTCGTGGAGTC | GCTGATGATCTTGAGGCTGTTGTC |
