# Supplementary material for: ERG-Associated lncRNA (ERGAL) Promotes the Stability and Integrity of Vascular Endothelial Barrier During Dengue Viral Infection via Interaction With miR-183-5p
Source: Front Cell Infect Microbiol. 2020 Sep 8;10:477. doi: 10.3389/fcimb.2020.00477 (PMC7506072; doi:10.3389/fcimb.2020.00477)
Supplement: Supplementary file 3 [file Table_3.DOCX]

**Table S3** | The sequence of si-ERGAL, miR-183-5p mimics and miR-183-5p inhibitor.

| **Gene name** | **Sequence** | |
| --- | --- | --- |
| si-ERGAL | GGACAAGTGTTGACTTTGA GATACTCATTAGACATCTA  GGAAAGTCTCCAATGTATA TATCAGCCTGCAGAATTATG  TTCCTGCATGAAATTACATC ACCTTCATCTCGAGAAAGGT | |
| miR-183-5p mimics | 5`-UAUGGCACUGGUAGAAUUCACU-3` | 5`-AGUGAAUUCUACCAGUGCCAUA-3` |
| miR-183-5p inhibitors | 5`-AGUGAAUUCUACCAGUGCCAUA-3` | |
