# Supplementary figures and images for: ERG-Associated lncRNA (ERGAL) Promotes the Stability and Integrity of Vascular Endothelial Barrier During Dengue Viral Infection via Interaction With miR-183-5p
Source: Front Cell Infect Microbiol. 2020 Sep 8;10:477. doi: 10.3389/fcimb.2020.00477 (PMC7506072; doi:10.3389/fcimb.2020.00477)

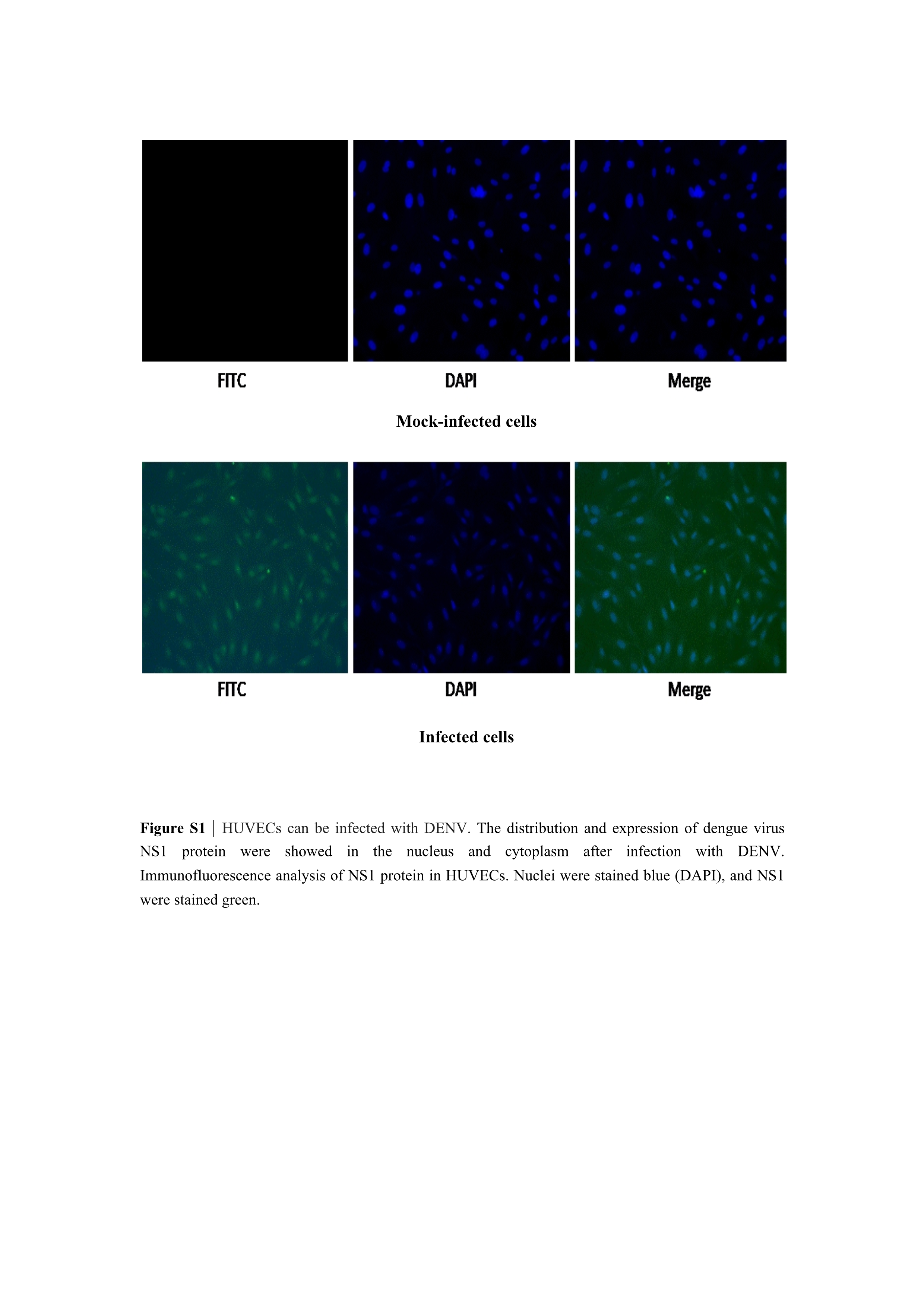

Supplement: Supplementary file 4 [file Image_1.tif]
